# Plant Antimicrobial Oligopeptides with Anticancer Properties as a Source of Biologically Active Peptides—An *In Silico* Study

**DOI:** 10.3390/ijms26189189

**Published:** 2025-09-20

**Authors:** Anna Jakubczyk, Kamila Rybczyńska-Tkaczyk, Anna Grenda

**Affiliations:** 1 Department of Biochemistry and Food Chemistry, University of Life Sciences in Lublin, 20-950 Lublin, Poland; anna.jakubczyk@up.edu.pl; 2 Department of Environmental Microbiology, University of Life Sciences in Lublin, 20-950 Lublin, Poland; 3 Department of Pneumonology, Oncology and Allergology, Medical University of Lublin, 20-059 Lublin, Poland; anna.grenda@umlub.edu.pl

**Keywords:** peptides, in silico, biologically active, anticancer peptides, antimicrobial peptides

## Abstract

Biologically active peptides can be obtained with various research methods, depending on the starting material, biological activity, and intended use. To use the most efficient method, it is worth combining in silico and in vitro experiments. Among the tools that can support an in silico analysis are databases such as the Antimicrobial Peptide Database (AMPD) or BIOPEP-UWM. The aim of this study was to make an in silico hydrolysis of peptides with anticancer properties selected from the AMP database, using pepsin, trypsin, and chymotrypsin. Most peptides obtained had properties inhibiting ACE and dipeptidyl peptidase IV activity. Among the resulting peptides, those with the sequence AR, CF, ER, TF, IY, ER, AW, GF, TW, SK and IM are potentially resistant to peptidase from microbial action. An analysis of the peptides’ characteristics showed that peptides with the sequence AR, EK, ER and SK are well-soluble in water and have high affinity for protein and ligand binding. Peptides with the sequence TF, IL and PF are unstable. Thermostable peptides are PGL, IL, GL, IY, VF, PL, IM and QL. The results of the study may be used to design in vitro experiments.

## 1. Introduction

Plant peptides are ubiquitously found in various parts of plants, including roots, seeds, flowers, stems, and leaves [1]. They can be extracted using several methods such as enzymatic hydrolysis, microbial fermentation, and chemical hydrolysis. Enzymatic hydrolysis is particularly favored due to its ability to yield specific products under mild conditions [2]. Biologically active plant peptides are small protein fragments derived from plants that exhibit a wide range of physiological activities. These peptides are produced through enzymatic processes and metabolic pathways in plants and are known for their significant medicinal and therapeutic potential. Plant-derived peptides have been shown to possess various biofunctional properties, including antibacterial, antioxidant, antihypertensive, anti-inflammatory, immunomodulatory, anticancer and antidiabetic ones [1,3,4].

Antibacterial plant-derived peptides (AMPs) are emerging as promising alternatives to conventional antibiotics due to their potent antimicrobial properties and lower likelihood of inducing resistance. Plant-derived AMPs exhibit broad-spectrum antimicrobial activity against various pathogens, including Gram-positive and Gram-negative bacteria, fungi, and viruses [5,6,7]. Combining AMPs with traditional antibiotics can enhance antibacterial efficacy and reduce the emergence of resistance. For instance, the combination of AMPs with erythromycin showed a synergistic effect, improving bacterial killing and reducing resistance development [8]. Moreover, AMPs have a lower propensity for inducing resistance compared to traditional antibiotics. This is partly due to their multiple mechanisms of action, which make it harder for bacteria to develop resistance [5,9]. These peptides are part of the plant’s innate defense system and exhibit broad-spectrum activity against various pathogens, including bacteria, fungi, viruses, and even cancer cells [10,11,12,13]. AMPs are typically small, cationic, and amphipathic molecules, often containing fewer than 100 amino acids. They can be rich in cysteine residues, forming disulfide bonds that contribute to their stability. Common types of AMPs include defensins, thionins, hevein-like peptides, knottins, snakins, lipid transfer proteins, and cyclotides [10,11,12,13,14]. Many AMPs act by permeabilizing bacterial membranes, leading to cell lysis. This can occur through various models, such as toroidal pore, barrel-stave, and carpet mechanisms. Some peptides interfere with bacterial metabolic processes or target specific intracellular components, further inhibiting bacterial growth. Certain AMPs induce reactive oxygen species (ROS) production within bacterial cells, causing oxidative damage and protein leakage [10,15,16,17]. AMPs hold significant promise as alternatives to conventional antibiotics, offering broad-spectrum antimicrobial activity and lower resistance potential. They are applied, for example, in medicine, agriculture and food industries, and the ongoing research aims at overcoming current challenges and enhancing the efficacy of such peptides [11,12,13,15,18]. AMPs are being explored for their potential in treating human infections, including those caused by multidrug-resistant bacteria. They are also being investigated for their anticancer and antiviral properties [11,19,20]. AMPs can enhance plant disease resistance and are used in developing bio-pesticides and transgenic plants to protect crops from pathogens [11,13]. Due to their antimicrobial properties, AMPs are considered for use in food preservation to prevent spoilage and extend shelf life [10,15].

There is a class of plant AMPs that have demonstrated anticancer activity, with some showing the ability to bind to cancer cell membranes and cause lysis. Modern personalized therapies are now available for oncological patients, which extend Progression-Free Survival (PFS) and Overall Survival (OS). In addition to the still widely used chemotherapy, molecularly targeted therapy and immunotherapies are becoming increasingly applicable [21,22].

Chemotherapy often causes serious side effects and leads to treatment resistance. Moreover, when using personalized therapies, there are different types of side effects, especially visible in immunotherapy targeted at immune checkpoints (ICI). Further, despite the effectiveness of modern cancer treatments, some patients experience resistance to therapy, the causes of which are not fully understood [23,24]. Because of this, there is an ongoing search for therapeutic methods that overcome primary or secondary resistance.

New compounds for cancer treatment with high efficacy and low toxicity are being sought. An example of such compounds are AMPs, which exert their anticancer effects primarily by destroying cell membranes; therefore, AMPs also have unique advantages in combating drug-resistant cancer cells [25]. Limitations in clinical use of AMPs, such as low proteolytic and chemical stability or salt sensitivity can be overcome by several methods, including stabilization, hybridization, cyclization, fragmentation, multimerization, alteration of amino acids, and conjugation or ligation [26]. Studies in animal models indicate that their anticancer mechanisms involve direct cytotoxicity, modulation of the host immune response, and direct impact on the tumor microenvironment, resulting in tumor regression, inhibition of metastasis, and improved survival rates [27].

Defensins, thionins, and cyclotides have antibacterial, antifungal, antiviral, and cytotoxic effects, which can be used to fight cancer cells. Peptides such as *Pyrularia*, *Viscotoxin*, *Ligatoxin*, and *β-Purothionin* have been shown to possess anticancer properties [28]. Cyclotides are known for their structural stability and the ability to interact with cell membranes. Their stability and tolerance to modifications of amino acid residues mean that they can be considered as scaffolds in the design of new anticancer drugs. The research by Flores-Alvarez et al. [29] demonstrates that defensin γ-thionin (*Capsicum chinense*) exhibits cytotoxic effects on K562 leukemia cells (IC50 = 290 μg/mL; 50.26 μM), while showing no toxicity toward human peripheral blood mononuclear cells. The findings revealed that γ-thionin did not alter the membrane potential, but it affected the mitochondrial membrane potential (∆Ψm) and triggered intracellular calcium release. Furthermore, γ-thionin promoted apoptosis in K562 cells without activating caspases 8 or 9. Notably, calpain activation was observed after one hour of treatment, indicating that γ-thionin induces caspase-independent apoptosis [29]. A number of plant-derived cyclotides have been described as demonstrating anticancer activity against breast, lung, cervical, intestinal or lymphoma cancer cells [30,31,32]. For example, modified Kalata B1 was tested as a vascular endothelial growth factor A (VEGF-A) antagonist [33]. A native cyclotide (MCoTI-II), on the other hand, has been tested for action against the BCR-ABL fusion protein (found as a product of the BCR/ABL gene fusion in leukemias) or the anti-CTLA-4 immune checkpoint, whose blockade with monoclonal antibodies is an immunotherapy registered and successfully used in immunogenic tumors [34,35]. This indicates the potential for using plant-derived peptides, with possible chemical modification, to develop drugs in the area of precision oncology. Moreover, it has been shown that Kalata B1 peptide can penetrate cells through both endocytosis and direct membrane translocation. Both pathways are initiated by targeting phosphatidylethanolamine phospholipids on the cell surface and by inducing changes in the cell membrane. This approach can be used to deliver drugs to cancer cells, which are characterized by a higher proportion of phosphatidylethanolamine phospholipids exposed on their surface [36]. Lunasin, a peptide found in soybean, wheat, and barley, has demonstrated anti-proliferative effects on cancer cells. Studies have found that lunasin inhibits non-small cell lung cancer cell proliferation by acting as an antagonist of αv integrin and a histone acetylation modulatory agent [37]. Lunasin alters the expression of G1-phase-specific components of the cyclin-dependent kinase complex, increases the level of cyclin-dependent kinase inhibitor 1B (p27Kip1), decreases the level of phosphorylated AKT (AKT Serine/Threonine Kinase 1) and, as a result, inhibits the phosphorylation of the retinoblastoma (RB) protein, simultaneously promoting its activation. Therefore, it is indicated that lunasin can inhibit non-small-cell lung cancer (NSCLC) proliferation by suppressing RB phosphorylation and inhibiting the cell cycle of tumor cells [38]. It is also asserted that lunasin is potentially applicable in treatment of metastatic, refractory and recurrent melanoma. It has been suggested that lunasin can reduce melanoma cell pools with stem cell properties. It acts by decreasing the phosphorylation of the activating intracellular kinases Focal Adhesion Kinase (FAK) and AKT, and reduces the acetylation of lysine residues in histones H3 and H4. In addition, it is indicated that lunasin’s ability to affect cancer-initiating cells is partly due to suppression of integrin signaling [39]. Research on the anticancer properties of peptides is now advancing, and is very promising if only in the form of developing supermolecular anticancer drugs that inhibit many of the abilities of cancerous cells [40]. Mechanisms related to self-organization and enzymatic regulation in cancer cells appear to be good targets for peptide-based potential anti-cancer therapies, whose efficacy may arise from reducing the communicative capacity of cancer cells and blocking signaling pathways.

Moreover, peptides can influence the tumor microenvironment, which participates in carcinogenesis at each of its stages. Through the use of appropriately prepared/modified peptides, the response to the applied registered treatment can be modulated, so they can serve as agents in supportive therapies [41]. Of course, the use of peptides in anti-cancer therapies is not without its challenges, including how to deliver them to the body or tumor cells. Therefore, nanotechnologies are being developed for the delivery of peptide molecules that will ensure efficient, precise and safe transport to their site of action [42].

There are many methods for obtaining biologically active peptides. The choice of a method depends on the starting material and the desired effect. Each method has its limitations and advantages. Among the methods for obtaining and characterizing biologically active peptides, there are in silico ones. In order to increase the accuracy and reliability of research on bioactive peptides, in silico studies are combined with in vitro and in vivo studies. This approach is particularly useful for identifying bioactive peptides from food sources and other biological materials. In silico methods are used to design peptides with high specificity and low toxicity for therapeutic purposes [43,44].

The aim of this study was to make an in silico hydrolysis of antimicrobial peptides with anticancer properties. Proteolytic enzymes of the digestive system, i.e., pepsin, trypsin and chymotrypsin, were selected for hydrolysis. The peptides obtained were tested in terms of resistance to the action of enzymes produced by gastrointestinal microorganisms and were analyzed for their physicochemical properties.

## 2. Results

### 2.1. Peptide Sequences After In Silico Hydrolysis with Gastrointestinal Track Enzymes

The peptides found in the Antimicrobial Peptide Database (AMPD) were in silico hydrolyzed under gastrointestinal tract conditions, and the all results are shown in Table S1. If any oligopeptide digestion result is not listed in the table, it means that no active fragments were found. The peptides obtained were shown according to their activity (Table 1). The resulting peptides consist of two and three amino acids. Most of the peptides were identified as angiotensin-converting enzyme (ACE) or dipeptidyl peptidase IV inhibitors. This is due to the amino acid composition of the obtained peptides, which determines their biological activity.

### 2.2. Peptide Sequences After Hydrolysis with Microbial Enzymes

In order to check whether the enzymes obtained are potentially resistant to the action of proteolytic enzymes produced by microorganisms in the small intestine, the peptides were hydrolyzed using oligopeptidase F and proteinase P1. The results are shown in Table 2. More than half of those peptides can be hydrolyzed by enzymes of microbial origin and can constitute a source of free amino acids for the body, especially since exogenous amino acids and proline, which is a conditionally exogenous amino acid, are released in a state of illness or stress. Those that remain intact can enter the bloodstream and be transported to their destination.

### 2.3. Characterization of the Peptides

The peptides were characterized according to physicochemical properties. Peptides with the sequences AR, EK, ER, PK, SK and QH were characterized by BI higher than 2.48, which suggested their high binding to the cell membrane or other protein receptors. Moreover, the peptides PK, SK, EK and ER are characteristic for a domain rich in basic residue, which promotes DNA/RNA binding. They are often found in nuclear localization sequences (NLS). This peptide signal sequence mediates the transfer of proteins from the cytoplasm into the nucleus. They are related, *inter alia*, to the action of importin α/β1, involved in the transport of functional proteins across the nuclear membrane. Importins cooperate with types of nuclear localization signals and take part in mechanisms of protein import into the nucleus. Thus, they are also involved in the transport of transcription factors, thereby participating in the regulation of gene expression. They may affect the activation of NF-κB (nuclear factor kappa-light-chain-enhancer of activated B cells), whether by participating directly in the regulation of gene expression by retention in the nucleus and by acting with other regulating proteins [45]. PTPN18 (Protein Tyrosine Phosphatase Non-Receptor Type 18) is a suppressor of breast cancer metastasis, thereby providing a potential effective antimetastatic therapeutic strategy. It has been proven that the nuclear (not the cytoplasmic) level is a factor beneficial in preventing metastasis, which is related to the expression and functioning of NLS-responsive importin β2 transporters [46]. Moreover, importins are being considered as new therapeutic targets in cancer [47].

All of the peptides have pI values of around or higher than 6. According to the results, it appears that only three peptides, with the sequence TF, IL or PF, are unstable. Some of the peptides—PGL, IL, GL, IY, VF, PL, IM and QL—are characterized by high thermostability. These peptides can be transported through the PEPT1/PEPT2 system; as short peptides, they can be transported by specific transporters in the small intestine. Most of the peptides obtained have a positive GRAVY index, meaning that they are hydrophobic. They can be components of membrane proteins. However, eight of the peptides are hydrophilic and can be components of soluble proteins. Their sequence is AR, EK, ER, PK, TW, PW, SK and QH. Only seven peptides, with the sequence AR, EK, ER, GK, PK SK and QH, are soluble in water. These peptides occur naturally in organisms and perform signaling, hormonal or neuromodulator functions.

## 3. Discussion

Biologically active peptides consist of 2 to 30 amino acid residues and can perform various biological and technological functions in the production of food, dietary supplements and drugs. There are many methods for obtaining biologically active peptides, and the choice of a method depends on the efficiency, application and source of these compounds. The most common methods include enzymatic hydrolysis of proteins, isolation of free peptides or de novo synthesis. Due to the fact that proteins are often the reserve material of plants, they are a good source of biologically active peptides. Currently, many experimental methods are preceded by data analysis and the use of in silico methods, enabled by various tools. This provides a basis for further research and is often considered a preliminary study or analysis of the research problem [48,49,50].

Plant-derived peptides obtained from the hydrolysis of plant proteins exhibit a wide range of stimulating activities, e.g., antioxidant, anti-inflammatory and immunomodulatory ones beneficial for human health. These peptides are bioactive compounds with diverse physiological functions, exhibiting a wide range of inhibitory activities against various enzymes, like inhibiting angiotensin-converting enzyme (ACE), renin, dipeptidyl peptidase-IV (DPP-IV) and peptidases, with significant potential for therapeutic applications in managing hypertension, metabolic disorders, neurodegenerative and inflammatory disorders [51,52,53,54].

A very important group of biologically active peptides consists of AMPs, which play a significant role in combating the activity of pathogenic microorganisms by being able to prevent their growth and colonization. They are natural biopolymers in organism protection. They exhibit broad activity against bacteria, viruses, and fungi [55]. Peptides are often multifunctional, which endows them with greater possibilities of use. It is true that their pharmacological use may be difficult due to pharmacokinetic disadvantages such as low proteolytic and chemical stability, high cytotoxicity and hemolytic activity, and salt sensitivity [26,27].

Nevertheless, plant-derived antimicrobial oligopeptides are a good source of peptides that exhibit various biological functions and which can be released in the digestive system. In this study, 57 oligopeptides with anticancer properties were selected from the AMP database and hydrolyzed *in silico* with gastrointestinal enzymes. AMP databases contain information on oligopeptide sequences with various properties, confirmed by scientific research. Due to the increasing need to identify compounds with anticancer properties, peptides that demonstrate anticancer properties and can also serve as a source of biologically active peptides were selected for this study. These peptides are inactive within the oligopeptide molecule and released under the influence of digestive enzymes. Oligopeptides were hydrolyzed with proteolytic enzymes of the gastrointestinal tract, i.e., pepsin, trypsin, and chymotrypsin, to simulate hydrolysis in the body. To obtain and design new foods or dietary supplements, it is necessary to understand the structure and characteristics of hydrolysis and absorption in an in silico [56] and then in vitro [57] model of the gastrointestinal tract so as to provide a theoretical basis for the action of bioactive compounds in the body. The majority of the di- and tripeptides obtained were those with antioxidant properties, inhibiting ACE activity, and dipeptidyl peptidase (Table 1). In vitro models offer several methods for simulating gastrointestinal conditions. These include pH changes, hydrolysis with single enzymes, sequences of digestive enzymes, and more complex models that incorporate, for example, intestinal peristalsis or gut microbiota. In studies on the absorption of bioactive compounds, experiments on intestinal epithelial cell lines are most commonly used [58]. The in silico methods offer cost-effective, less time-consuming high-throughput alternatives to traditional in vitro and in vivo methods. Many studies confirm the reliability of in silico studies in in vitro research. McFarland et al. (2024) [59] performed in silico and in vitro digestibility analysis of a sweet protein derived from sweet truffle (*Mattirolomyces terfezioides*). For in silico digestibility, PeptideCutter was employed using the enzyme pepsin at pH 1.3, along with trypsin and chymotrypsin, to predict the protein digestion products. In vitro digestibility was determined using a commercial proteolytic enzyme kit with pepsin, trypsin, and chymotrypsin [59]. In silico digestion of the protein indicated high digestibility (43 peptide fragments were obtained), which was confirmed by experimental studies. In vitro mass spectral analysis confirmed the presence of all predicted tryptic fragments. The correlation between the in silico and in vitro results confirms the validity and reliability of the computational model in predicting the production of peptides that may exhibit biological activity.

Studies on the protein stability of the Serendipity berry plant (*Dioscoreophyllum cumminsii* (Stapf) Diels) also support the in silico approach. Both in vitro and in silico results indicate >90% digestibility, and the in silico results indicate 15 pepsin cleavage sites and 15 trypsin cleavage sites, resulting in an average fragment size of 3.43 amino acids. Therefore, it can be concluded that the protein tested is highly digestible. The small peptide fragments predicted by the in silico model (averaging approximately 3.5 amino acids) suggest that the protein will most likely be efficiently broken down and available for absorption, which correlates well with the high digestibility observed in the study using proteolytic enzymes [60]. In silico studies do not take into account many factors, such as sample preparation, hydrolysis time, or intestinal peristalsis. However, they allow for preliminary conclusions as to whether a given research material is suitable for research and whether it is worth further characterization. Other studies also confirm comparable in silico and in vitro results. In studies of bean proteins as a functional component, the BIOPEP-UWM database was used to determine in silico protein digestibility, using pepsin, trypsin, and chymotrypsin. In the experimental studies, pepsin, pancreatic juices, amylase, and bile acid were used. The in silico digestion results showed that peptides with lengths of two to five amino acids were released in the largest amount (51.13%), followed by free amino acids (34.09%), and finally, peptides longer than five amino acids were present in the smallest amount (14.77%). In vitro digestibility ranged from 48.2% to 63.6%, depending on the protein content and whether the samples were cooked or uncooked [61].

Elisha et al. studied cow’s and human milk proteins as a potential source of peptides with ACE- and dipeptidyl peptidase IV-inhibiting properties. In this case, in silico studies also confirmed experimental studies using proteolytic enzymes from the digestive system [62].

Therefore, it is advisable to conduct in silico studies, which significantly shortens the time and is less time-consuming and labor-intensive. It also allows for the appropriate selection of research material, which can contribute to increased effectiveness of in vitro and in vivo studies.

Plant-derived peptides have garnered significant attention due to their potent antioxidant properties, which can protect against oxidative damage and related diseases. These peptides are primarily obtained through the enzymatic hydrolysis of plant proteins, which breaks down the proteins into smaller, bioactive fragments [63,64,65,66,67]. Plant-derived antioxidant peptides are sourced from various plants, including cereals, legumes, oilseeds, fruits, and vegetables [52,68]. These peptides exhibit strong antioxidant activities by scavenging free radicals, chelating metal ions, and modulating antioxidant enzymes. They help reduce intracellular reactive oxygen species and enhance the body’s defense mechanisms [66,67,69]. The antioxidant activity is influenced by the peptides’ amino acid composition and sequence. Peptides with hydrophobic amino acids (e.g., L or V) and those containing sulfur-containing (C or M) or aromatic amino acids (F, W or Y) tend to have stronger antioxidant properties [67]. These findings correspond well with the results obtained in our study, where the antioxidant peptides were indicated as TW, IY, AW and PW (Table 1). The antioxidant effects are often linked to the peptides’ability to modulate key oxidative stress pathways, such as Keap1-Nrf2-ARE, MAPK, NF-κB, and PI3K/AKT/mTOR. They also influence the expression of pro- and anti-apoptotic proteins and antioxidant enzymes [66,69].

Plant-derived antihypertensive peptides are gaining attention as natural supplements for managing hypertension owing to their efficacy, safety, and non-toxic side effects. These peptides are fragments of plant proteins that exhibit biological activity, particularly in inhibiting enzymes involved in blood pressure regulation [51,70,71,72]. Many plant-derived peptides inhibit the angiotensin-converting enzyme (ACE), which plays a crucial role in blood pressure regulation by promoting vasodilation through increased nitric oxide production [51,52,71]. Some peptides also inhibit renin, another enzyme involved in the renin-angiotensin system (RAS), which helps control blood pressure. These peptides may also interact with angiotensin II receptors, further contributing to their antihypertensive effects [71]. Some plant-derived peptides enhance the biosynthesis of nitric oxide (NO) in the vascular endothelium. NO is a vasodilator that helps relax blood vessels, thereby reducing blood pressure [71]. Certain plant-derived peptides can modulate the Renin–angiotensin–aldosterone system (RAAS), which plays a crucial role in blood pressure regulation [53].

The relationship between the amino acid composition and ACE inhibition has not been fully elucidated. Dipeptides including L, Q, P, M or A in their structure have been shown to have strong ACE inhibitory activity. Moreover, tripeptides with L, C or P also exhibit strong ACE inhibitory activity. It should be noted that peptides with hydrophobic C-terminus amino acids are potent ACE inhibitors. Additionally, peptides with a molecular mass below 2 kDa tend to have higher ACE inhibitory activity due to better absorption and bioavailability [73]. In our study, the peptides obtained with ACE inhibitory active, such as PGL, CF, TF, IL, GL, VF, AW, PL and GL, also had a hydrophobic C-terminus residue (Table 1). Similar results were obtained by Elisha et al., where peptides obtained from cow’s and human milk lactoferrin exhibiting diepeptyl peptidase IV inhibitory activity had the sequences GF, GL, AF, SF, and GAL. In the case of bovine, the peptides PF, PL, GL, VF, and GAL showed potential. Furthermore, the sequences YL, CL, CAL, GR, CR, and CSTSPL were common to both lactoferrin from cow and human milk [62].

Plant-derived peptides interact with the human immune system through various mechanisms, including immunomodulation and anti-inflammatory activities. Plant-derived peptides exhibit significant anti-inflammatory properties through various mechanisms. These peptides can modulate inflammatory responses by interacting with specific cellular pathways and molecules [74,75,76]. Plant peptides can reduce the levels of pro-inflammatory cytokines such as IL-6, IL-1β, and TNF-α [75,77,78,79]. This reduction helps in mitigating inflammation and its associated symptoms. These peptides promote the polarization of macrophages to the M2 phenotype, which is associated with anti-inflammatory effects. This shift in a macrophage phenotype helps in resolving inflammation. Many plant-derived peptides inhibit the NF-κB signaling pathway, which plays a crucial role in the inflammatory response. By down-regulating NF-κB, these peptides reduce the expression of inflammatory mediator enzymes, such as cyclooxygenase (COX-2) and nitric oxide synthase (iNOS) [78,80]. Moreover, they can inhibit lipoxygenase (LOX) involved in arachidonic acid metabolism [80]. Plant peptides interfere with the metabolism of arachidonic acid, thereby reducing the production of pro-inflammatory eicosanoids. This mechanism is crucial in controlling inflammation at the molecular level. Some plant peptides can bind to lipopolysaccharides (LPS), neutralizing their inflammatory effects. This binding reduces the recognition of LPS by immune cells, thereby attenuating the inflammatory response [78].

To test the potential use of the peptides obtained as compounds that could be transported in the small intestine to their target sites, their resistance to microbial enzymes was tested in silico. The peptides were hydrolyzed using oligopeptidase F and proteinase P1. This process was also conducted using the BIOPEP databases. The results indicate that not all peptides with specific functions are stable to the action of these enzymes (Table 2). Unstable peptides, on the other hand, can be a good source of free amino acids essential for proper body function [81]. Furthermore, these peptides primarily release exogenous amino acids (essential amino acids), which are not produced in the body and must be supplied through food [82]. It should also be noted that these peptides release proline, which is considered a relatively essential amino acid, the demand for which increases during periods of illness or severe stress [83,84]. This creates the possibility of producing new preparations or dietary supplements that would contain these peptides.

Proteolytic instability is one of the key features of peptides and peptide-containing products, which determines their use in the production of food, dietary supplements or pharmacological compounds. Research is underway to increase their resistance to peptidases/proteinases or to enhance their stability in plasma or serum. These methods most often involve chemical or physical modification, and are often labor-intensive and cost-prohibitive. Therefore, understanding the chemical properties of peptides that influence their degradation facilitates modifications intended to increase their stability. One of the important features of peptides in this context is the determination of the peptide half-life (t ½), which is usually quite short and ranges from 2 to 20 min. Some studies show, however, that the t ½ of these compounds can be even more than 300 min [85]. Extending the half-life of biologically active peptides increases their stability, bioavailability, and applicability. There is no database of peptide half-lives, so these values must be determined experimentally [86].

Moreover, one of the peptides (IL) obtained has neuropeptide properties. Neuropeptides are involved in numerous biological processes, including neurotransmission, neuromodulation, and hormonal regulation. They control functions like cardiovascular activity, energy homeostasis, reproduction, growth, behavior, and stress response [54].

One of the key stages in peptide research is physicochemical analysis, which employs various techniques and methods to determine properties of peptides and possible applications in various fields, including diagnostics, food technology, biotechnology, drug development, and proteomics [87,88]. In this study, the following peptide characteristics were determined: the Boman index, net charge, isoelectric point, instability index, aliphatic index, GRAV, and water solubility (Table 3). By determining the physicochemical properties of peptides, we can also infer their potential bioavailability. Peptides with short chains consisting of two or three amino acids will demonstrate greater bioavailability than those containing more amino acids. Moreover, peptides characterized by high solubility potentially have higher bioavailability than those that are insoluble [86,89].

The Boman index is a measure used to predict the potential biological activity of peptides, particularly their ability to bind to membranes or other proteins as receptors. It is expressed in kcal/mol, and it is calculated from the sum of free energies of the amino acid residues in a peptide sequence. Molecules have high binding potential if the BI is higher than 2.48 [90]. This is supported by research results concerning the arginine-rich cationic peptide SM-985 from teosinte (*Zea mays* spp. *mexicana*), whose microbial activity has been verified for six species, and its Boman index was 5.19 kcal/mol [91]. In our study, peptides with the sequence AR, EK, ER, PK, SK, and QH have high potential for binding into a protein or receptor. It should be noted that ten amino acids—A, Cys, Gly, His, Ile, Lys, Leu, Pro, Arg, and Val—are often observed in AMPs [92]. As can be seen from the presented data, peptides with the sequence PGL, IL, GL, IY, VF, PL, IM and QL are characterized by high thermostability because the instability index is higher than 100. This is because these dipeptides contain hydrophobic amino acids. Thermostable peptides can have many applications, especially in chemical analysis, food and pharmaceutical industries, and also in agriculture and environmental protection. Thermostable peptides can be engineered to respond to environmental stimuli, such as temperature, pH, and ionic strength, making them versatile for different applications [93]. Moreover, thermostable AMPs have been designed to maintain their activity under high temperatures and varying pH levels. These peptides can be used in food preservation to inhibit bacterial growth, ensuring food safety and extending shelf life [94]. Additionally, compounds can pose analytical challenges due to their in vitro stability. In order to address this issue, their instability index is determined.

In this study, only two peptides were determined as unstable. They are the peptides with the sequence TF, IL and PF. These dipeptides are composed of hydrophobic amino acids and are poorly soluble in water, so they may not be easy research material in an aqueous environment. The GRAVY index may define the overall hydropathy of a peptide. In this study, the peptides with the sequence AR, EK, ER, PK, TW, PW, SK and QH were characterized by a negative GRAVY index, which indicated their hydrophilic character, meaning that they interact well with water and are likely to be soluble in aqueous environments. Understanding this index helps to predict protein behavior in different environments and can aid in such applications as drug design and protein engineering. Filleria et al. ([95] reported that hydrophobicity may help to increase the antioxidant activity. In their study, the linear sequence of each peptide is observed as well as their variations and a putative relationship with the antioxidant activity. P2 is the only peptide that presents a hydrophobic character according to the GRAVY parameter and has the highest ASA_H_. P1, P5 and P8 are hydrophilic in agreement with their proportion of ASA_P_ [95]. Understanding the physicochemical properties of peptides can contribute to their increased use in anticancer therapy. Many methods exist for selectively targeting and destroying cancer cells, both proliferative and non-proliferative ones, while bypassing healthy cells. One of these methods is using lipid membranes as an alternative therapeutic target. By disrupting the cancer cell membrane or allowing various compounds to penetrate the cell membrane, they can induce selective cancer cell death. During cancer development, cell membranes undergo changes. It is worth noting that the cell membrane of cancer cells differs from that of non-cancerous cells. The lipid bilayer of healthy cell membranes is characterized by an asymmetric distribution of lipids. The use of cell membranes as a potential therapeutic target is related to the anticancer activity of host defense peptides. A key role is played by cationic amphipathic membrane peptides, which directly target cancer cells by binding to their negatively charged surfaces through electrostatic interactions. Peptides integrate into the hydrophobic core of the cancer cell membrane through polar and nonpolar peptide-lipid interactions. Some peptides at nanomolar concentrations can penetrate the cell membrane and cause cancer cell death. However, at higher concentrations (in the micromolar range), they disrupt the lipid bilayer, often killing cells within an hour [96,97]. That is why it is so important to know the structure and properties of biologically active peptides in order to use them in various therapeutic activities.

## 4. Materials and Methods

The material for the study was composed of 57 plant peptides (Table 4) with anticancer properties that were selected from the Antimicrobial Peptide Database (https://aps.unmc.edu/home access date 10 September 2025).

### 4.1. In Silico Enzymatic Hydrolysis

#### 4.1.1. Hydrolysis with Gastrointestinal Track Enzymes

The selected peptides were hydrolyzed using BIOPEP-UWM™ database tools [98] with pepsin (EC 3.4.23.1), trypsin (EC 3.4.21.4), and chymotrypsin (EC 3.4.21.1).

#### 4.1.2. Hydrolysis with Microbial Enzymes

Peptides with biological activity were in silico hydrolyzed with oligopeptidase F and proteinase P1 (EC 3.4.21.96). These enzymes were produced by gastrointestinal track microorganisms.

### 4.2. Analysis of the Physicochemical Characteristics of Bioactive Peptides

The physicochemical characteristics of peptides were predicted with online tools. The Boman index was estimated by the Antimicrobial Peptide Designer (https://aps.unmc.edu/, access data 10 September 2025); net charge and water solubility were calculated using the peptide property calculator available on https://pepcalc.com/. access data 10 September 2025. The Protein IQ tool (https://proteiniq.io/tools#protein-tools access data 10 September 2025) was used to estimate theoretical isoelectric point (TpI), instability index, aliphatic index and grand average of hydropathicity index (GRAVY).

## 5. Conclusions

In silico methods have become essential tools in the study and prediction of the biological properties of peptides. These computational approaches offer significant advantages in terms of cost and time efficiency compared to traditional experimental methods. In this study, 57 plant peptides with anticancer properties selected from the Antimicrobial Peptide Database (https://aps.unmc.edu/home, access on 10 September 2025) were hydrolyzed using BIOPEP-UWM™ database tools with pepsin (EC 3.4.23.1), trypsin (EC 3.4.21.4), and chymotrypsin (EC 3.4.21.1). Most peptides obtained after in silico hydrolysis had properties inhibiting ACE and dipeptidyl peptidase IV activity. Some peptides have previously unknown properties, like inhibitory activities towards DPP III (TF, PF and GF), lactocepin (PL), neprilysin (AR), acylaminoacyl peptidase and TPP II (AR). Moreover, one of the peptides (IL) has neuropeptide properties. The use of these peptides that are resistant to the action of proteolytic enzymes of microbial origin seems promising. Further research and development are needed to overcome bioavailability challenges and validate the clinical efficacy of peptides. Research should be based on in vitro studies, i.e., enzymatic hydrolysis of the selected peptides using pepsin, trypsin, and chymotrypsin. Subsequent purification and characterization of the peptides should follow, prior to their application in the design of food products, dietary supplements, or therapeutic formulas. Further in vivo studies are necessary for this purpose. The presented research results serve as a prelude to further analyses and can provide an overview of the research material for obtaining and characterizing biologically active peptides. Owing to their origin and physicochemical properties, biologically active peptides may find application in the creation of new foods or dietary supplements with therapeutic properties. Many peptides act directly as drugs, mimicking natural hormones, enzymes, or ligands (e.g., insulin, GLP-1 analogues, calcitonin). Others can be attached to drugs or nanoparticles to improve transport to tissues or receptors (e.g., RGD-motif peptides for targeting integrins in tumors). Some peptides (cell-penetrating peptides, CPPs) facilitate drug transport across biological barriers, such as the blood–brain barrier or cell membranes. Peptides can act as excipients, stabilizing proteins, preventing their aggregation, or improving solubility in formulations.

## Figures and Tables

**Table 1:** Peptides obtained after pepsin, trypsin and chymotrypsin *in silico* hydrolysis.

Activity	Sequence	Source (AP ID)
ACE inhibitor	AR	AP00236
	PGL	AP00984
	CF	AP00984
	EK	AP00984, AP02329
	TF	AP00979
	IL	AP00979
	GL	AP01026, AP01124, AP01784, AP01785, AP01806, AP01807, AP02332, AP02657
	IY	AP01277, AP01278, AP01279, AP01280, AP01281, AP01282, AP01284, AP01328
	ER	AP01280, AP01282, AP01284, AP01328
	VF	AP01343
	AW	AP01805
	PL	AP02657
	GK	AP02328
	GF	AP05050
Antioxidative	PK	AP00236
	TW	AP00236
	IY	AP01277, AP01278, AP01279, AP01280, AP01281, AP01282, AP01284, AP01328
	AW	AP01805
	PW	AP01986, AP01988
Dipeptidyl peptidase IV inhibitor	PK	AP00236, AP01278, AP01279, AP01280, AP01281, AP01282, AP01284, AP01328
	TW	AP00236
	EK	AP00984, AP02329
	IL	AP00979
	TF	AP00979
	GL	AP01026, AP01124, AP01784, AP01785, AP01806, AP01807, AP02332, AP02657
	SK	AP01036, AP01123, AP01124, AP01774, AP01777, AP01808, AP01813, AP01983, AP02329, AP05050
	VF	AP01343
	AW	AP01805
	PF	AP01985
	PW	AP01986, AP01988
	IM	AP02329
	QH	AP02329
	QL	AP02329
	PL	AP02657
	GF	AP05050
Stimulating	IL	AP00979
Renin inhibitor	TF	AP00979
Dipeptidyl peptidase III inhibitor	TF	AP00979
	PF	AP01985
	GF	AP05050
Neuropeptide	IL	AP00979
ACE2 inhibitor	PF	AP01985
Xaa-pro inhibitor	PL	AP02657
Lactocepin inhibitor	PL	AP02657
Acylaminoacyl peptidase inhibitor	GF	AP05050
Tripeptidyl peptidase II inhibitor	GF	AP05050
Neprilysin inhibitor	AR	AP00236

**Table 2:** Peptides resistant to hydrolysates of enzymes from microorganism.

Peptide Sequence	Oligopeptidase F Action	Proteinase P1 Action
AR	AR	AR
PGL	PGL	P
		G
		L
CF	CF	CF
EK	EK	EK
TF	TF	TF
IL	IL	I
		L
GL	GL	G
		L
IY	IY	IY
ER	ER	ER
VF	VF	V
		F
AW	AW	AW
PL	PL	P
		L
GF	GF	GF
GK	GK	GK
PK	PK	P
		K
TW	TW	TW
PW	PW	P
		W
SK	SK	SK
PF	PF	P
		F
IM	IM	IM
QH	QH	Q
		H
QL	QL	Q
		L

**Table 3:** Physicochemical characteristic of peptides.

Peptides Sequence	Index Boman (kcal/mol)	Net Charge	Theoretical pI	Instability Index	Aliphatic Index	GRAVY	Water Solubility
AR	6.55	1	10.55	5.0	50	−1.35	good
PGL	−1.95	0	6.10	6.67	130.00	0.60	poor
CF	−2.13	−0.1	5.92	5.0	0	2.65	poor
EK	6.18	0	6.41	5.0	0	−3.70	good
TF	−0.2	0	6.10	66.70	0	1.05	poor
IL	−4.92	0	6.10	101.30	390.00	4.15	poor
GL	−2.93	0	6.10	5.0	195.00	1.70	poor
IY	−2.39	0	6.09	5.0	195.00	1.60	poor
ER	10.86	0	6.41	5.0	0	−4.00	good
VF	−3.51	0	6.10	5.0	145.00	3.50	poor
AW	−2.07	0	6.10	5.0	50	0.45	poor
PL	−2.46	0	6.10	5.0	195.00	1.10	poor
GF	−1.96	0	6.10	5.0	0	1.20	poor
GK	2.30	0	6.70	−3745	0	−2.15	good
PK	2.77	1	9.70	5.0	0	−2.75	good
TW	0.11	0	6.10	−70.15	0	−0.80	poor
PW	−1.16	0	6.10	−9.40	0	−1.25	poor
SK	4.47	1	9.70	5.0	0	−2.35	good
PF	−1.49	0	6.10	101.30	0	0.60	poor
IM	−3.63	0	6.10	5.0	195.00	3.20	poor
QH	5.09	0.1	7.55	5.0	0	−3.35	good
QL	0.31	0	6.10	5.0	195.00	0.15	poor

**Table 4:** The sequences of plant antimicrobial peptides with anticancer properties.

No	APD ID	Name	Source	Sequence
1	AP00236	Pyrularia thionin	Nuts, *Pyrularia pubera*	KSCCRNTWARNCYNVCRLPGTISREICAKKCDCKIISGTTCPSDYPK
2	AP00532	Lunatusin	Lima bean *Phaseolus lunatus* L.	KTCENLADTFRGPCFATSNC
3	AP00553	Sesquin	Ground bean seeds, *Vigna sesquipedalis*	KTCENLADTY
4	AP00984	TPP3	Tomato, *Lycopersicon esculentum*	QICKAPSQTFPGLCFMDSSCRKYCIKEKFTGGHCSKLQRKCLCTKPC
5	AP00979	NaD1	ornamental tobacco flowers, *Nicotiana alata*	RECKTESNTFPGICITKPPCRKACISEKFTDGHCSKILRRCLCTKPC
6	AP01026	Varv peptide A	*Viola arvensis, Viola odorata*, *Viola tricolor*, *Viola baoshanensi*, *Viola yedoensis*, and *Viola biflora*	GLPVCGETCVGGTCNTPGCSCSWPVCTRN
7	AP01031	Varv peptide F	*Viola arvensis*	GVPICGETCTLGTCYTAGCSCSWPVCTRN
8	AP01036	Cycloviolacin O^2^	*Viola odorata*	GIPCGESCVWIPCISSAIGCSCKSKVCYRN
9	AP01121	Vibi E	Alpine violet *Viola biflora*	GIPCAESCVWIPCTVTALIGCGCSNKVCYN
10	AP01123	Vibi G	Alpine violet *Viola biflora*	GTFPCGESCVFIPCLTSAIGCSCKSKVCYKN
11	AP01124	Vibi H	Alpine violet *Viola biflora*	GLLPCAESCVYIPCLTTVIGCSCKSKVCYKN
12	AP01277	Viscotoxin A3	The European mistletoe, *Viscum album* L.	KSCCPNTTGRNIYNACRLTGAPRPTCAKLSGCKIISGSTCPSDYPK
13	AP01278	Viscotoxin 1-Ps	The European mistletoe, *Viscum album* L.	KSCCPNTTGRNIYNTCRFGGGSREVCARISGCKIISASTCPSDYPK
14	AP01279	Viscotoxin A1	*Viscum album* L. seeds	KSCCPNTTGRNIYNTCRLTGSSRETCAKLSGCKIISASTCPSNYPK
15	AP01280	Viscotoxin C	The Asiatic *Viscum album* ssp. Coloratum ohwi	KSCCPNTTGRNIYNTCRFAGGSRERCAKLSGCKIISASTCPSDYPK
16	AP01281	Viscotoxin A2	*Viscum album* L.	KSCCPNTTGRNIYNTCRFGGGSRQVCASLSGCKIISASTCPSDYPK
17	AP01282	Viscotoxin B	*Viscum album* L.	KSCCPNTTGRNIYNTCRLGGGSRERCASLSGCKIISASTCPSDYPK
18	AP01284	Viscotoxin B2	*Viscum coloratum* (Kom.) Nakai	KSCCKNTTGRNIYNTCRFAGGSRERCAKLSGCKIISASTCPSDYPK
19	AP01342	Cn-AMP1	Green coconut water, *Cocos nucifera*	SVAGRAQGM
20	AP01343	Cn-AMP2	Green coconut water, *Cocos nucife*	TESYFVFSVGM
21	AP01774	Cliotide T1	*Clitoria ternatea*	GIPCGESCVFIPCITGAIGCSCKSKVCYRN
22	AP01775	Cliotide T2	*Clitoria ternatea*	GEFLKCGESCVQGECYTPGCSCDWPICKKN
23	AP01776	Cliotide T3	*Clitoria ternatea*	GLPTCGETCTLGTCYVPDCSCSWPICMKN
24	AP01777	Cliotide T4	*Clitoria ternatea*	GIPCGESCVFIPCITAAIGCSCKSKVCYRN
25	AP01784	Vaby A	Africa, the Ethiopian highlands, *Viola abyssinica*	GLPVCGETCAGGTCNTPGCSCSWPICTRN
26	AP01785	Vaby D	Africa, the Ethiopian highlands, *Viola abyssinica*	GLPVCGETCFGGTCNTPGCTCDPWPVCTRN
27	AP01805	Cr-ACP1	Seeds, *Cycas revoluta*	AWKLFDDGV
28	AP01806	Viba 15	*Viola philippica*	GLPVCGETCVGGTCNTPGCACSWPVCTRN
29	AP01807	Viba17	*Viola philippica*	GLPVCGETCVGGTCNTPGCGCSWPVCTRN
30	AP01808	Viphi A	*Viola philippica*	GSIPCGESCVFIPCISSVIGCACKSKVCYKN
31	AP01809	Viphi D	*Viola philippica*	GIPCGESCVFIPCISSVIGCSCSSKVCYRN
32	AP01810	Viphi E	*Viola philippica*	GSIPCGESCVFIPCISAVIGCSCSNKVCYKN
33	AP01811	Viphi F	*Viola philippica*	GSIPCGESCVFIPCISAIIGCSCSSKVCYKN
34	AP01812	Viphi G	*Viola philippica*	GSIPCEGSCVFIPCISAIIGCSCSNKVCYKN
35	AP01813	Mram 8	*Viola philippica*	GIPCGESCVFIPCLTSAIDCSCKSKVCYRN
36	AP01983	Psyle A	*Psychotria leptothyrsa*	GIACGESCVFLGCFIPGCSCKSKVCYFN
37	AP01984	Psyle E	*Psychotria leptothyrsa*	GVIPCGESCVFIPCISSVLGCSCKNKVCYRD
38	AP01985	Psyle C	*Psychotria leptothyrsa*	KLCGETCFKFKCYTPGCSCSYPFCK
39	AP01986	ChaC1	Hybrid peptide of melittin and protamine	GDACGETCFTGICFTAGCSCNPWPTCTRN
40	AP01987	ChaC2	*Chassalia chartacea*	GIPCAESCVWIPPCTITALMGCSCKNNVCYNN
41	AP01988	ChaC4	*Chassalia chartacea*	GASCGETCFTGICFTAGCSCNPWPTCTRN
42	AP01989	ChaC7	*Chassalia chartacea*	IPCGESCVWIPCITAIAGCSCKNKVCYT
43	AP01990	ChaC8	*Chassalia chartacea*	AIPCGESCVWIPCISTVIGCSCSNKVCYR
44	AP01991	ChaC10	*Chassalia chartacea*	GEYCGESCYLIPCFTPGCYCVSRQCVNKN
45	AP01992	ChaC11	*Chassalia chartacea*	IPCGESCVWIPCISGMFGCSCKDKVCYS
46	AP02325	Cliotide T7	*Clitoria ternatea*	GIPCGESCVFIPCTVTALLGCSCKDKVCYKN
47	AP02326	Cliotide T10	*Clitoria ternatea*	GVPCAESCVWIPCTVTALLGCSCKDKVCYLN
48	AP02327	Cliotide T12	*Clitoria ternatea*	GIPCGESCVYIPCTVTALLGCSCKDKVCYKN
49	AP02328	Cliotide T19	*Clitoria ternatea*	GSVIKCGESCLLGKCYTPGCTCSRPICKKD
50	AP02329	Lunasin	*Glycine max*	SKWQHQQDSCRKQLQGVNLTPCEKHIMEKIQGRGDDDDDDDDD
51	AP02332	PaDef	Avocado fruit, *Persea americana* var. drymifolia	CETPSKHFNGLCIRSSNCASVCHGEHFTDGRCQGVRRRCMCLKPC
52	AP02340	Cyclosaplin	Somatic seedlings, *Santalum album* L.	RLGDGCTR
53	AP02657	Vigno 5	*Viola ignobilis*	GLPLCGETCVGGTCNTPGCSCGWPVCVRN
54	AP02659	DC1	*Hedyotis diffusa*	GAFLKCGESCVYLPCLTTVVGCSCQNSVCYRD
55	AP02660	DC2	*Hedyotis diffusa*	GAVPCGETCVYLPCITPDIGCSCQNKVCYRD
56	AP02661	DC3	*Hedyotis diffusa*	GTSCGETCVLLPCLSSVLGCTCQNKRCYKD
57	AP05050	Hyen D	*Hybanthus enneaspermus*	GFPCGESCVYIPCFTAAIGCSCKSKVCYKN

## Data Availability

The raw data supporting the conclusions of this article will be made available by the authors on request.

## Supplementary Materials

The following supporting information can be downloaded at: https://www.mdpi.com/article/10.3390/ijms26189189/s1.

## Abbreviations

The following abbreviations are used in this manuscript:

TPP II	Tripeptidyl peptidase II
DPP III	Dipeptidyl peptidase-III
LPS	Lipopolysaccharides
LOX	Lipoxygenase
iNOS	Nitric oxide synthase
COX-2	Cyclooxygenase
RAAS	Renin–angiotensin–aldosterone system
NO	Nitric oxide
PTPN18	Protein tyrosine phosphatase non-receptor type 18
NF-κB	Nuclear factor kappa-light-chain-enhancer of activated B cells
RAS	Renin-angiotensin system
NLS	Nuclear localization sequences
ACE2	Angiotensin-converting enzyme 2
ACE	Angiotensin-converting enzyme
FAK	Focal adhesion kinase
NSCLC	Non-small-cell lung cancer
RB	Retinoblastoma
p27Kip1	Cyclin-dependent kinase inhibitor 1B
MCoTI-II	A native cyclotide
AMPD	Antimicrobial peptide database
AMPs	Antibacterial plant-derived peptides
ROS	Reactive oxygen species
VEGF-A	Vascular endothelial growth factor A
