# Supplementary material for: Plant Antimicrobial Oligopeptides with Anticancer Properties as a Source of Biologically Active Peptides—An In Silico Study
Source: Int J Mol Sci. 2025 Sep 20;26(18):9189. doi: 10.3390/ijms26189189 (PMC12471097; doi:10.3390/ijms26189189)
Supplement: Supplementary file 1 [file ijms-26-09189-s001.zip › ijms-3830818-supplementary.pdf]

Table S1. Peptide sequence after *in silico* hydrolysis with pepsin, trypisin and chymptrypisin

AP ID	Peptide ID	Sequence	Location	Activity
AP00236	7742	AR	[9-10]	ACE inhibitor
	8459	TW	[7-8]	antioxidative
	8858	PK	[46-47]	dipeptidyl peptidase IV inhibitor
	8913	TW	[7-8]	dipeptidyl peptidase IV inhibitor
	10771	PK	[46-47]	Antioxidative
	10907	AR	[9-10]	Neprilysin inhibitor
AP00984	7507	PGL	[11-13]	ACE inhibitor
	7751	CF	[14-15]	ACE inhibitor
	7840	EK	[27-28]	ACE inhibitor
	8558	EK	[27-28]	dipeptidyl peptidase IV inhibitor
AP00979	8185	TF	[9-10]	ACE inhibitor
	8323	IL	[37-38]	Stimulating
	8802	IL	[37-38]	dipeptidyl peptidase IV inhibitor
	8900	TF	[9-10]	dipeptidyl peptidase IV inhibitor
	9079	IL	[37-38]	ACE inhibitor
	9471	TF	[9-10]	Renin inhibitor
	9486	TF	[9-10]	DPP-III inhibitor
	10455	IL	[37-38]	Neuropeptide
AP01026	7599	GL	[1-2]	ACE inhibitor
	8561	GL	[1-2]	dipeptidyl peptidase IV inhibitor
AP01036	8894	SK	[24-25]	dipeptidyl peptidase IV inhibitor
AP01123	8894	SK	[25-26]	dipeptidyl peptidase IV inhibitor
AP01124	7599	GL	[1-2]	ACE inhibitor
	8561	GL	[1-2]	dipeptidyl peptidase IV inhibitor
	8894	SK	[25-26]	dipeptidyl peptidase IV inhibitor
AP01277	3383	IY	[12-13]	ACE inhibitor
	7873	IY	[12-13]	antioxidative
	8858	PK	[45-46]	dipeptidyl peptidase IV inhibitor
AP01278	3383	IY	[12-13]	ACE inhibitor
	7873	IY	[12-13]	antioxidative
	8858	PK	[45-46]	dipeptidyl peptidase IV inhibitor
AP01279	3383	IY	[12-13]	ACE inhibitor
	7873	IY	[12-13]	antioxidative
	8858	PK	[45-46]	dipeptidyl peptidase IV inhibitor
AP01280	3383	IY	[12-13]	ACE inhibitor
	7873	IY	[12-13]	antioxidative
	8858	PK	[45-46]	dipeptidyl peptidase IV inhibitor
	9944	ER	[24-25]	ACE inhibitor
AP01281	3383	IY	[12-13]	ACE inhibitor
	7873	IY	[12-13]	antioxidative
	8858	PK	[45-46]	dipeptidyl peptidase IV inhibitor

AP01282	3383	IY	[12-13]	ACE inhibitor
	7873	IY	[12-13]	antioxidative
	8858	PK	[45-46]	dipeptidyl peptidase IV inhibitor
	9944	ER	[24-25]	ACE inhibitor
AP01284	3383	IY	[12-13]	ACE inhibitor
	7873	IY	[12-13]	antioxidative
	8858	PK	[45-46]	dipeptidyl peptidase IV inhibitor
	9944	ER	[24-25]	ACE inhibitor
AP01328	3383	IY	[12-13]	ACE inhibitor
	7873	IY	[12-13]	antioxidative
	8858	PK	[45-46]	dipeptidyl peptidase IV inhibitor
	9944	ER	[24-25]	ACE inhibitor
AP01343	3384	VF	[6-7]	ACE inhibitor
	8917	VF	[6-7]	dipeptidyl peptidase IV inhibitor
AP01774	8894	SK	[24-25]	dipeptidyl peptidase IV inhibitor
AP01777	8894	SK	[24-25]	dipeptidyl peptidase IV inhibitor
AP01784	7599	GL	[1-2]	ACE inhibitor
	8561	GL	[1-2]	dipeptidyl peptidase IV inhibitor
AP01805	7543	AW	[1-2]	ACE inhibitor
	8460	AW	[1-2]	antioxidative
	8695	AW	[1-2]	dipeptidyl peptidase IV inhibitor
AP01806	7599	GL	[1-2]	ACE inhibitor
	8561	GL	[1-2]	dipeptidyl peptidase IV inhibitor
AP01807	7599	GL	[1-2]	ACE inhibitor
	8561	GL	[1-2]	dipeptidyl peptidase IV inhibitor
AP01808	8894	SK	[25-26]	dipeptidyl peptidase IV inhibitor
AP01813	8894	SK	[24-25]	dipeptidyl peptidase IV inhibitor
AP01983	8894	SK	[22-23]	dipeptidyl peptidase IV inhibitor
AP01985	8854	PF	[22-23]	dipeptidyl peptidase IV inhibitor
	9505	PF	[22-23]	dipeptidyl peptidase III inhibitor
	10460	PF	[22-23]	ACE2 inhibitor
AP01986	8190	PW	[22-23]	antioxidative
	8865	PW	[22-23]	dipeptidyl peptidase IV inhibitor
AP01988	8190	PW	[22-23]	antioxidative
	8865	PW	[22-23]	dipeptidyl peptidase IV inhibitor
AP02328	7611	GK	[13-14]	ACE inhibitor
AP02329	7840	EK	[28-29]	ACE inhibitor
	8558	EK	[28-29]	dipeptidyl peptidase IV inhibitor
	8803	IM	[26-27]	dipeptidyl peptidase IV inhibitor
	8872	QH	[4-5]	dipeptidyl peptidase IV inhibitor
	8874	QL	[13-14]	dipeptidyl peptidase IV inhibitor
	8894	SK	[1-2]	dipeptidyl peptidase IV inhibitor
AP02332	7599	GL	[10-11]	ACE inhibitor

	8561	GL	[10-11]	dipeptidyl peptidase IV inhibitor
AP02657	7513	PL	[3-4]	ACE inhibitor
	7599	GL	[1-2]	ACE inhibitor
	8561	GL	[1-2]	dipeptidyl peptidase IV inhibitor
	8638	PL	[3-4]	dipeptidyl peptidase IV inhibitor
	10462	PL	[3-4]	xaa-pro inhibitor
	10463	PL	[3-4]	lactocepin inhibitor
AP05050	7591	GF	[1-2]	ACE inhibitor
	8782	GF	[1-2]	dipeptidyl peptidase IV inhibitor
	8894	SK	[24-25]	dipeptidyl peptidase IV inhibitor
	9488	GF	[1-2]	DPP-III inhibitor
	10583	GF	[1-2]	Acylaminoacyl peptidase inhibitor
	10584	GF	[1-2]	Tripeptidyl peptidase II inhibitor
